# Functional single nucleotide polymorphisms within the cyclin-dependent kinase inhibitor 2A/2B region affect pancreatic cancer risk

**DOI:** 10.18632/oncotarget.10935

**Published:** 2016-07-29

**Authors:** Daniele Campa, Manuela Pastore, Manuel Gentiluomo, Renata Talar-Wojnarowska, Juozas Kupcinskas, Ewa Malecka-Panas, John P. Neoptolemos, Willem Niesen, Pavel Vodicka, Gianfranco Delle Fave, H. Bas Bueno-de-Mesquita, Maria Gazouli, Paola Pacetti, Milena Di Leo, Hidemi Ito, Harald Klüter, Pavel Soucek, Vincenzo Corbo, Kenji Yamao, Satoyo Hosono, Rudolf Kaaks, Yogesh Vashist, Domenica Gioffreda, Oliver Strobel, Yasuhiro Shimizu, Frederike Dijk, Angelo Andriulli, Audrius Ivanauskas, Peter Bugert, Francesca Tavano, Ludmila Vodickova, Carlo Federico Zambon, Martin Lovecek, Stefano Landi, Timothy J. Key, Ugo Boggi, Raffaele Pezzilli, Krzysztof Jamroziak, Beatrice Mohelnikova-Duchonova, Andrea Mambrini, Franco Bambi, Olivier Busch, Valerio Pazienza, Roberto Valente, George E. Theodoropoulos, Thilo Hackert, Gabriele Capurso, Giulia Martina Cavestro, Claudio Pasquali, Daniela Basso, Cosimo Sperti, Keitaro Matsuo, Markus Büchler, Kay-Tee Khaw, Jakob Izbicki, Eithne Costello, Verena Katzke, Christoph Michalski, Anna Stepien, Cosmeri Rizzato, Federico Canzian

**Affiliations:** ^1^ Department of Biology, University of Pisa, Pisa, Italy; ^2^ Genomic Epidemiology Group, German Cancer Research Center (DKFZ), Heidelberg, Germany; ^3^ Department of Digestive Tract Diseases, Medical University of Lodz, Lodz, Poland; ^4^ Department of Gastroenterology, Lithuanian University of Health Sciences, Kaunas, Lithuania; ^5^ Institute for Health Research Liverpool Pancreas Biomedical Research Unit, University of Liverpool, Liverpool, United Kingdom; ^6^ Department of General, Visceral and Transplantation Surgery, Heidelberg University Hospital, Heidelberg, Germany; ^7^ Institute of Experimental Medicine, Czech Academy of Science, Prague, Czech Republic; ^8^ Institute of Biology and Medical Genetics, 1^st^ Medical Faculty, Charles University, Prague, Czech Republic; ^9^ Digestive and Liver Disease Unit, S. Andrea Hospital, ‘Sapienza’ University of Rome, Rome, Italy; ^10^ Department for Determinants of Chronic Diseases (DCD), National Institute for Public Health and the Environment (RIVM), Bilthoven, The Netherlands; ^11^ Department of Epidemiology and Biostatistics, The School of Public Health, Imperial College London, London, United Kingdom; ^12^ Department of Social & Preventive Medicine, Faculty of Medicine, University of Malaya, Kuala Lumpur, Malaysia; ^13^ Department of Basic Medical Sciences, Laboratory of Biology, Medical School, National and Kapodistrian University of Athens, Athens, Greece; ^14^ Oncological Department Massa Carrara Azienda USL Toscana Nord Ovest, Carrara, Italy; ^15^ Gastroenterology and Gastrointestinal Endoscopy Unit, Vita-Salute San Raffaele University, IRCCS San Raffaele Scientific Institute, Milan, Italy; ^16^ Division Epidemiology and Prevention, Aichi Cancer Center Research Institute, Nagoya, Japan; ^17^ Institute of Transfusion Medicine and Immunology, German Red Cross Blood Service Baden-Württemberg – Hessen gGmbH, Medical Faculty Mannheim, Heidelberg University, Mannheim, Germany; ^18^ Laboratory of Toxicogenomics, National Institute of Public Health, Prague, Czech Republic; ^19^ Laboratory of Pharmacogenomics, Biomedical Center, Faculty of Medicine in Pilsen, Charles University in Prague, Pilsen, Czech Republic; ^20^ ARC-Net Research Centre, and Department of Diagnostics and Public Health University and Hospital Trust of Verona, Verona, Italy; ^21^ Department of Gastroenterology, Aichi Cancer Center Hospital, Nagoya, Japan; ^22^ Division of Cancer Epidemiology, German Cancer Research Center (DKFZ), Heidelberg, Germany; ^23^ Department of General, Visceral and Thoracic Surgery, University Medical Center Hamburg-Eppendorf, Hamburg, Germany; ^24^ Division of Gastroenterology and Research Laboratory, IRCCS Scientific Institute and Regional General Hospital “Casa Sollievo della Sofferenza”, San Giovanni Rotondo, Italy; ^25^ Department of Gastroenterological Surgery, Aichi Cancer Center Hospital, Nagoya, Japan; ^26^ Department of Pathology, Academic Medical Centre, Amsterdam, The Netherlands; ^27^ Biomedical Center, Faculty of Medicine in Pilsen, Charles University in Prague, Prague, Czech Republic; ^28^ Department of Medicine - DIMED, University of Padova, Padova, Italy; ^29^ Department of Surgery I, Faculty of Medicine and Dentistry, Palacky University Olomouc and University Hospital Olomouc, Olomouc, Czech Republic; ^30^ Epidemiology Unit Nuffield Department of Population Health University of Oxford, Oxford, UK; ^31^ Division of General and Transplant Surgery, Pisa University Hospital, Pisa, Italy; ^32^ Pancreas Unit, Department of Digestive System, Dant'Orsola-Malpighi Hospital, Bologna, Italy; ^33^ Department of Hematology, Institute of Hematology and Transfusion Medicine, Warsaw, Poland; ^34^ Department of Oncology, Faculty of Medicine and Dentistry, Palacky University Olomouc and University Hospital Olomouc, Olomouc, Czech Republic; ^35^ Blood Transfusion Service, Azienda Ospedaliero Universitaria Meyer, Florence, Italy; ^36^ Department of Surgery, Academic Medical Centre, Amsterdam, The Netherlands; ^37^ Colorectal Unit, First Department of Propaedeutic Surgery, Athens Medical School, National and Kapodistrian University of Athens, Athens, Greece; ^38^ Department of Surgery, Oncology and Gastroenterology-DiSCOG, University of Padova, Padova, Italy; ^39^ Department of Laboratory Medicine, University-Hospital of Padova, Padova, Italy; ^40^ Division of Molecular Medicine, Aichi Cancer Center Research Institute, Nagoya, Japan; ^41^ Clinical Gerontology Unit, Addenbrooke's Hospital, School of Clinical Medicine, University of Cambridge, Cambridge, UK; ^42^ Laboratory of Clinical, Transplant Immunology and Genetics, Copernicus Memorial Hospital, Lodz, Poland; ^43^ Department of Translational Research and New Technologies in Medicine and Surgery, University of Pisa, Pisa, Italy

**Keywords:** pancreatic cancer, CDKN2A, single nucleotide polymorphisms, miRSNP, association study

## Abstract

The *CDKN2A* (*p16*) gene plays a key role in pancreatic cancer etiology. It is one of the most commonly somatically mutated genes in pancreatic cancer, rare germline mutations have been found to be associated with increased risk of developing familiar pancreatic cancer and *CDKN2A* promoter hyper-methylation has been suggested to play a critical role both in pancreatic cancer onset and prognosis. In addition several unrelated SNPs in the 9p21.3 region, that includes the *CDNK2A, CDNK2B* and the *CDNK2B-AS1* genes, are associated with the development of cancer in various organs. However, association between the common genetic variability in this region and pancreatic cancer risk is not clearly understood. We sought to fill this gap in a case-control study genotyping 13 single nucleotide polymorphisms (SNPs) in 2,857 pancreatic ductal adenocarcinoma (PDAC) patients and 6,111 controls in the context of the Pancreatic Disease Research (PANDoRA) consortium. We found that the A allele of the rs3217992 SNP was associated with an increased pancreatic cancer risk (OR_het_=1.14, 95% CI 1.01-1.27, p=0.026, OR_hom_=1.30, 95% CI 1.12-1.51, p=0.00049). This pleiotropic variant is reported to be a mir-SNP that, by changing the binding site of one or more miRNAs, could influence the normal cell cycle progression and in turn increase PDAC risk. In conclusion, we observed a novel association in a pleiotropic region that has been found to be of key relevance in the susceptibility to various types of cancer and diabetes suggesting that the *CDKN2A/B* locus could represent a genetic link between diabetes and pancreatic cancer risk.

## INTRODUCTION

The majority of pancreatic cancer patients die within a year of diagnosis [1]. The poor prognosis is caused by various factors, including the lack of appropriate markers for early detection, the aggressiveness of the disease and the dearth of effective treatment possibilities available. One of the best strategies to reduce the mortality of the disease is to improve early diagnosis, and it is, therefore, important to identify individuals at high risk in the population and subject them to enhanced surveillance.

Only a few epidemiologic risk factors have been established for pancreatic cancer, including cigarette smoking, heavy alcohol intake, diabetes mellitus, obesity, chronic pancreatitis and family history of pancreatic cancer [2, 3]. Even less is known about the genetic contribution to the disease, since only a rather small number of susceptibility *loci* have been identified through genome-wide association studies (GWAS) [4–9] and confirmed by follow-up studies [10]. Moreover it has been shown that a small proportion of pancreatic tumors arises as a result of high penetrance germline mutations in genes such as *BRCA1, BRCA2*, *p16/CDKN2A*, *STK11/LKB, ATM, PALB2*, and DNA mismatch repair genes, usually in the context of familial cancer syndromes [2, 3, 11–15]. However, the very low frequency of those mutations cannot explain the bulk of genetic susceptibility to pancreatic cancer.

There are compelling epidemiologic and molecular evidences pointing to a key role for the *CDKN2A* gene in pancreatic cancer etiology. *CDKN2A* is one of the most commonly somatically mutated genes in pancreatic cancer [16], rare germline mutations have been found to be associated with increased risk of developing familiar pancreatic cancer [15, 17], and also *CDKN2A* promoter hyper-methylation has been suggested to play a critical role both in pancreatic cancer onset and prognosis [18]. Additionally, the 9p21.3 region, that includes in addition to *CDKN2A* also *CDKN2B* and *CDKN2B-AS1*, is pleiotropic and several polymorphic variants in the region spanning the three genes are susceptibility markers for several cancer types [19–24]. In addition Li and colleagues performed an association study and meta-analysis across multiple cancers corroborating the pleiotropic role of the locus [25]. Several polymorphic variants in the region are also associated with type two diabetes mellitus (T2DM), which is a predisposing factor for pancreatic cancer, suggesting a possible role of the 9p21.3 region as a genetic link between the two diseases [26–28]. The pleiotropic role of the variants of this region is probably due to the central importance of the genes situated in it in cell cycle regulation. For example *CDKN2A* codes, by alternative splicing, for the two oncosuppressors p16INK4a and p14ARF [29, 30]. Despite all the hints pointing towards an association between common genetic variability in the *CDKN2A/B* gene region and pancreatic cancer risk no one has attempted to directly relate them so far. We sought to fill this gap in a case-control study genotyping 13 single nucleotide polymorphisms (SNPs) in the context of the PANcreatic Disease ReseArch (PANDoRA) consortium.

## RESULTS

### Data filtering and quality control

The characteristics of the population enrolled in the study are shown in Table 1. All analyzed SNPs were in HWE in controls (P>0.0038) with the exception of rs3731246 in the controls from Mannheim (Germany) and Southern Italy and rs2811710 in the controls from Mannheim. The populations not respecting the HWE were not included in the statistical analyses for the relevant SNPs. Starting from a population of 9,796 subjects, 828 subjects with a call rate <75% were removed after genotyping, leaving 8,968 for further analysis. The average SNP call rate was 96% with a minimum of 79.95% (rs3731246) and a maximum of 99.19% (rs3218009). The analysis of the random duplicate samples showed a concordance rate of 99.68%. For the Japanese cases it was not possible to correctly genotype rs3731246 and therefore this SNP was not used in the risk analysis for the Japanese population. Supplementary Table S1 shows the call rate and the HWE equilibrium for each SNP.

**Table 1:** Characteristics of the studied population

	Cases	Controls	Total
**Median age (25%-75% percentiles)**	65 (57-72)	57 (48-64)	
**Gender**			
Males	1637	3371	5008
Females	1182	2677	3859
Unknown	38	63	101
**Geographic origin**			
Germany	1066	2282	3348
Czech Republic	251	518	769
Greece	80	175	255
Italy Center	480	549	1029
Italy North	361	595	956
Italy South	114	500	614
Lithuania	57	190	247
Poland	87	335	422
Netherlands	117	102	219
UK	99	176	275
Japan	145	689	834
**Total**	**2857**	**6111**	**8968**

### SNP main effects

We analyzed separately Caucasian and Japanese individuals. For the individuals of Caucasian origin we observed that 6 SNPs showed a statistically significant association (p<0.05) with increased or decreased PDAC risk. The strongest association with an increased risk of PDAC was observed with the A allele of rs3217992 SNP (OR_het_=1.14, 95% CI 1.01-1.27, p=0.026, OR_hom_=1.30, 95% CI 1.12-1.51, p=0.00049, unadjusted p-trend =0.0002, adjusted p-trend= 0.32). Other, less significant associations were observed with rs3731249, rs2811708, rs3731211, and rs1063192. The frequencies and distributions of the genotypes (for the Caucasian group) and the OR for the association of each polymorphism with PDAC are described in Table 2. In the Japanese population the G allele of rs2811708 was associated with a decreased risk of PDAC (OR_het_=0.58, 95% CI 0.36-0.95, p=0.029) and the A allele of rs1063192 was associated with a decreased risk of PDAC (OR_het_=0.49, 95% CI 0.30-0.80, p=0.005, p-trend=0.03). The frequencies and distribution of the Japanese genotypes and the OR for the association of each polymorphism with PDAC are described in Table 3.

**Table 2:** Association between the selected SNPs and PDAC risk in the Caucasian population

SNP	Alleles^a^	Cases^b^			Controls^b^			MM vs Mm		p	MM vs mm		p	p-trend adj	p-trend unadj	SNP Annotation^d^
	(M/m)	MM	Mm	mm	MM	Mm	mm	OR^c^	95% CI^c^		OR	95% CI				
rs3731257	C/T	1,362	1,082	211	2,567	2,007	354	0.99	0.89-1.10	0.83	1.07	0.88-1.31	0.476	0.419	0.30	5′ flanking
rs11515	C/G	1,875	700	50	3,568	1,297	118	1.00	0.89-1.13	0.92	0.70	0.48-1.01	0.058	0.70	0.81	3′ UTR
rs2518719	G/A	2,057	542	46	3,779	1,124	100	0.90	0.79-1.01	0.09	0.97	0.66-1.42	0.87	0.15	0.03	Intronic
rs3731249	C/T	2,496	159	5	4,140	341	9	0.75	0.60-0.92	0.007	0.64	0.19-2.07	0.45	0.14	0.01	Missense
rs3731246	C/G	1,814	450	37	1,842	515	30	0.85	0.73-0.99	0.04	1.10	0.65-1.85	0.72	0.39	0.32	Intronic
rs2811708	T/G	1,382	894	145	2,770	1,985	359	0.90	0.80-1.00	0.057	0.76	0.61-0.95	0.015	0.03	0.009	Intronic
rs3731239	C/T	1,066	1,193	313	2,237	2,365	673	1.06	0.96-1.19	0.25	0.99	0.84-1.18	0.92	047	0.82	Intronic
rs3731211	A/T	1,387	997	190	2,220	1,701	380	0.96	0.86-1.08	0.51	0.75	0.62-0.92	0.007	0.29	0.02	Intronic
rs2811710	T/C	1,087	1,207	333	1,413	1,636	504	1.00	088-1.12	0.97	0.88	0.74-1.05	0.17	0.26	0.08	Intronic
rs3218009	C/G	2,188	437	23	4,071	888	66	0.96	0.84-1.10	0.54	0.75	0.45-1.24	0.26	0.52	0.04	Intronic
rs3217992	G/A	821	1,332	504	1,807	2,543	868	1.14	1.01-1.27	0.026	1.30	1.12-1.51	0.0005	0.32	0.0002	3′UTR
rs1063192	G/A	1,011	1,219	341	1,756	2,291	722	0.89	0.80-0.99	0.047	0.82	0.70-0.97	0.019	0.55	0.009	3′UTR
rs3217986	C/A	2,243	392	17	4,511	810	28	0.97	0.84-1.12	0.71	1.48	0.78-2.82	0.23	0.14	0.89	3′UTR

a M = major allele (i.e. more common in controls); m = minor allele (less common in controls).

b Numbers may not add up to 100% due to genotyping failure, DNA depletion or covariate missing values.

c Odds ratio (95% confidence interval).

d as shown in Haploreg

All analyses were adjusted for age at diagnosis/age at recruitment, gender and country of origin.

**Table 3:** Association between the selected SNPs and PDAC risk in the Japanese population

SNP	Alleles^a^	Cases^b^			Controls^b^			MM vs Mm		p	MM vs mm		p	p-trend adj	p-trend unadj
	(M/m)	MM	Mm	mm	MM	Mm	mm	OR	95% CI		OR	95% CI			
rs3731257	C/T	24	68	49	128	336	204	1.10	0.66-1.83	0.71	1.30	0.76-2.23	0.33	0.41	0.32
rs11515	C/G	131	7	0	643	17	0	1.97	0.80-4.85	0.14		-		1.00	0.12
rs2518719	G/A	145	0	0	667	0	0		-			-		-	-
rs3731249	C/T	136	0	0	683	0	0		-			-		-	-
rs2811708	T/G	101	23	5	452	176	18	0.58	0.36-0.95	0.029	1.23	0.45-3.41	0.68	0.59	0.15
rs3731239	C/T	109	27	2	507	164	14	0.76	0.48-1.21	0.25	0.66	0.15-2.96	0.59	0.24	0.22
rs3731211	A/T	97	38	9	429	218	20	0.77	0.51-1.16	0.21	2.01	0.89-4.55	0.09	0.85	0.97
rs2811710	T/C	82	52	9	376	272	36	0.87	0.59-1.27	0.48	1.15	0.53-2.47	0.73	0.41	0.81
rs3218009	C/G	143	0	0	687	0	0		-			-			
rs3217992	G/A	28	44	50	128	289	218	0.70	0.41-1.17	0.17	1.05	0.63-1.75	0.85	0.44	0.59
rs1063192	G/A	99	22	9	432	198	32	0.49	0.30-0.80	0.005	1.23	0.57-2.66	0.60	0.03	0.11
rs3217986	C/A	130	14	0	622	66	0	1.00	0.55-1.85	0.98				0.06	0.96

a M = major allele (i.e. more common in controls); m = minor allele (less common in controls).

b Numbers may not add up to 100% due to genotyping failure, DNA depletion or covariate missing values.

c Odds ratio (95% confidence interval).

All analyses were adjusted for age at diagnosis/age at recruitment, gender and country of origin.

### Possible functional effects

We used several bioinformatic tools to predict possible functional relevance for the SNPs showing the most significant associations. Using Genevar, we observed that the C allele of rs3217992 was associated with increased expression of the interferon alpha 4 (*IFNA4*) gene (P=0.047). This association, however, was not below the threshold suggested by Genevar for significance (P<10^−3^). GTEx did not show any signifcant eQTL for any of the SNPs associated with pancreatic cancer risk. RegulomeDB showed a score of 5, indicating the possible presence of a transcription factor binding motif or a DNase sensitivity peak for rs2811708, rs3217992 and rs1063192 and a score of 4 suggesting the presence of a transcription factor binding motif and a DNase sensitivity peak for rs3731211 and rs3731249. HaploReg did not suggest any relevant signals for the significant SNPs.

## DISCUSSION

Several unrelated SNPs in the 9p21.3 region, that includes the *CDNK2A, CDNK2B* and the *CDNK2B-AS1* genes, are associated with the development of cancer in various organs [19–21, 23, 24, 31–33] and with T2D [26–28]. In this study we have performed an in depth analysis of the region and found several promising associations between the common genetic variability and the disease onset. However, considering the correction for multiple testing only rs3217992 showed a statistically significant association with increased risk of developing PDAC.

This finding is interesting for two reasons. The first is that rs3217992 shows a plethora of associations with several human traits such as primary open-angle glaucoma [34], aggressive periodontitis, chronic periodontitis [35] and myocardial infarction [36]. These phenotypes are very different from each other, but all the studies, including the one presented here, have in common the fact that is always the A allele to be associated with the increased risk of the disease. This observation strongly suggests a pleiotropic role for rs3217992 and also highlights that the polymorphisms alters the function of the protein in a way that affects the related phenotypes. In recent years several pleiotropic SNPs have been identified to be associated with multiple human phenotypes or multiple cancer types. These pleiotropic stretches of DNA that are densely packed with risk alleles have been defined Nexus regions [37] and intensely studied in relation to cancer risk. PDAC has at least another such region in the *TERT-CLPTM1L* locus as widely demonstrated by several authors [6, 9, 38].

The second reason is that in addition to the strong statistical association and the putative pleiotropic effect, rs3217992 SNP lies in a miRNA target region (miR-138-2-3p). In a very recent study Ghanbari and collaborators showed, using cardiac cell lines, that the rs3217992 SNP might have an effect on the miRNA-mRNA interactions [39]. Specifically, the G allele increased the miRNA-dependent regulation of *CDKN2B*. Given the role of CDKN2B in the cell cycle this result seems counterintuitive since the A allele is associated with increased risk in our study and in others as mentioned before. The 9p21.3 locus has, however, a very complex regulation, and it is possible that the binding between the G allele and miR-138-2-3p might be specific for the cardiac tissue. Moreover the 9p21.3 locus hosts several genes that are key cell cycle regulators and therefore the fact that rs3217992 changes the binding site of one or more miRNAs could influence the normal cell cycle progression and in turn increase PDAC risk. There are growing evidences that mir-SNPs could be involved in various pathologies including cancer and diabetes given their ability to affect gene regulation. However, the functional significance of this SNP needs thorough scrutiny in the pancreatic tissue to address its possible role in PDAC susceptibility.

Another finding of potential significance is the association between the A allele of rs1063192 with decreased PDAC risk. This SNP also lies in the *CDKN2B* 3′UTR, is a putative mir-SNP and shows a weak linkage disequilibrium (LD) with rs3217992 (r^2^=0.474 in CEU and r^2^=0.170 CHBJPT, 1000 Genomes Project). Li and colleagues found this SNP to be associated with esophageal squamous cell carcinoma [25], while in a recent report [40] the A allele of rs1063192 was found to be associated with a decreased risk of developing gestational diabetes mellitus (GDM) suggesting a direct genetic link between diabetes and pancreatic cancer though the genetic variants analyzed in both studies.

The strongest association we observed in the Japanese population was between the A allele of rs1063192 and a decreased PDAC risk. This finding gives rise to two considerations: the first is that even though the association does not reach the threshold of significance considering Bonferroni's correction, it is unlikely to be a false finding giving the fact that it was found in two different populations. The second is that since in the Caucasian and in the Japanese the leading SNP is not the same it is possible that the real causal variant is yet untyped and mildly in LD with rs1063192 and rs3217992.

We also observed an association close to statistical significance (taking into account multiple testing) between the missense SNP rs3731249 and PDAC susceptibility. This variation is predicted by PolyPhen to be possibly damaging with a score of 0.487 and has been widely investigated in childhood acute lymphocytic leukemia [23, 24, 41], once again highlighting the pleiotropic nature of this genomic region. Through dbGaP we could perform a GWAS look-up for the results of our candidate SNPs. We found results only for Caucasians (PanScan2, [6]) and only for three of the significant SNPs rs2811708, rs3217992 and rs1063192. All the SNPs showed ORs that were in the same direction with our results but none showed a statistically significant association, the best was p=0.08 for rs1063192. In 2 out of 3 populations (PANDoRA Japanese and PanScan) tested the best association was observed for rs1063192 and in the last population (PANDoRA Caucasians) for rs3217992. The two SNPs are in weak LD. This suggests the hypothesis that another variant (possibly a low frequency/rare one) may be associated with both, that was not typed in either population and that has an LD pattern with rs1063192 and rs3217992 that varies in the different populations. The difference in the results between PANDoRA and PanScan may be explained by the fact that in PANDoRA the strongest statistical association was observed for the rare homozygous carriers compared with the common allele carriers suggesting a recessive model of inheritance, while in the PanScan data only the allelic model is shown. Additionally, the results from PanScan arise partly from a prospective cohort while ours are from a case-control study. Although the biological explanation of this phenomenon is difficult to understand, discrepancies between the two study designs have been observed several times in different neoplastic diseases [4, 21, 42, 43].

One of the major strengths of this study is its size, since with a total of 8,968 subjects this is one the largest genetic analysis of pancreatic cancer risk published to-date. Additionally, our selection of SNPs provides an extensive coverage of genetic variability in the regions of interest. In addition we could analyze simultaneously two ethnic groups, which helps to generalize the findings. A possible limitation of the study is that patients and controls in PANDoRA were recruited in various centers across Europe and therefore there might be some population-based differences. Moreover we did not include rare variants and therefore we cannot exclude to have missed associations due to alleles with a MAF<0.05.

In conclusion we observed several novel associations in a pleiotropic region that has been found to be of key relevance in the susceptibility to various types of cancer and diabetes, confirming a key role for *CDKN2A/B* in pancreatic cancer and suggesting a possible involvement of the common genetic variability at this locus in PDAC risk.

## MATERIALS AND METHODS

### Study population

In this study 2,857 pancreatic ductal adenocarcinoma (PDAC) patients and 6,111 controls were collected in nine countries (Germany, Czech Republic, Greece, Italy, Lithuania, Poland, Netherlands, UK and Japan) belonging to the PANDoRA consortium that has been described in detail elsewhere [44]. Briefly, cases were retrospectively collected and are defined by a confirmed diagnosis of PDAC. Controls were recruited in the same hospitals, or at least geographical region from where the cases were recruited. British and Dutch controls (N=176 and 102, respectively) were selected from healthy volunteers recruited from the general population in the European Prospective Investigation on Cancer (EPIC), an ongoing prospective cohort being carried out in ten European countries [45]. The German controls used were partly blood donors from the blood transfusion center in Mannheim and partly healthy volunteers selected among EPIC subjects collected in Heidelberg. Patients provided written informed consent and the study was approved by Ethical Review Board of the University of Heidelberg (Medizinische Facultaet Heidelberg) the study was performed in accordance with Declaration of Helsinki.

### SNP selection

To survey the common genetic variability in the *CDKN2A*/*B* locus we selected tagging SNPs using the Tagger tool of the Haploview software using the Caucasian population in the HapMap web site (International HapMap Project, version 28; http://www.hapmap.org) as reference (http://www.broad.mit.edu/mpg/haploview/; http://www.broad.mit.edu/mpg/tagger/). We considered a region centered on the *CDKN2A* gene region and we added 5k bp at 5′ and 5k at 3′ of the gene resulting in a window of around 40k base pair. We used a pairwise tagging method with a minimum r^2^ of 0.8 and a minor frequency allele of 0.05 to select tagging SNPs inside the region. The first tagging SNP at the 5′ end, rs3731257, is situated at 21956221 (Hg18) while the last tagging SNP at the 3′ end, rs3217986, is situated at 21995330 (Hg18). In addition we added two functional SNPs (rs1063192 and rs3217992) that are putative miR-SNPs, i.e. predicted to alter the binding of one or more microRNAs to their target. The final selection resulted in 13 SNPs in the 9p21.3 region. We checked if the tagging selection used was valid also for the Asian ethnicities and we obtained that the tagging set for Chinese and Japanese is a subset of the one used for Caucasians.

### Sample preparation and genotyping

For each sample DNA was extracted from whole blood or from paraffin-embedded pancreatic tissues of patients and controls using the AllPrep Isolation Kit (Qiagen, Hilden, Germany) or the Qiagen-mini kit (Qiagen, Hilden, Germany), according to the manufacturer's protocol. Genotyping was performed using TaqMan (ABI, Applied Biosystems, Foster City, CA, USA) and KASPar (KBioscence, Hoddesdon, UK) technologies. The order of DNA samples was randomized on plates in order to ensure that similar numbers of cases and controls were analyzed in each batch. Detection was performed using an ABI PRISM 7900 HT or Viia7 sequence detection system with SDS 2.2 or Viia7 software (Applied Biosystems, Foster City, CA, USA). Genotyping for British and Dutch controls was performed in the context of a genome-wide association study using the Human 660W-Quad BeadChip array according to manufacturer's instructions (Illumina, San Diego, CA, USA). For quality control, duplicates of 10% of the samples were interspersed throughout the plates. In addition, we discarded all the samples that had a call rate < 75%.

### Statistical analysis

The observed genotype frequencies of all SNPs in the control subjects were tested for deviation from Hardy–Weinberg equilibrium (HWE) using Pearson's chi-square test. The association between the genotypes of all polymorphisms and PDAC risk was estimated using an unconditional logistic regression computing odds ratios (OR), 95% confidence intervals (95% CIs) and p values. The more common allele among the controls was assigned as the reference category and the co-dominant model inheritance model was assessed. All analyses were adjusted for age, gender and geographic origin (among the Europeans countries). European and Japanese individuals were kept separate in the analyses. All analyses were adjusted for multiple testing using the Bonferroni correction whereby a significance threshold of 0.0038 (0.05/13) was set.

### Bioinformatic analysis

To assess the possible functional relevance for the SNPs showing the most significant associations with risk of developing PDAC several bioinformatic tools were used. RegulomeDB (http://regulome.stanford.edu/)[46] and HaploReg v2B [47] were used to identify the regulatory potential of the region nearby each SNP. Genevar (http://www.sanger.ac.uk/resources/software/genevar/) [48] and GTEx (http://www.gtexportal.org/home/)[49] were used to identify potential associations between the SNP and expression levels of nearby genes (eQTL).

## SUPPLEMENTARY MATERIALS TABLE


