# Supplementary material for: Functional single nucleotide polymorphisms within the cyclin-dependent kinase inhibitor 2A/2B region affect pancreatic cancer risk
Source: Oncotarget. 2016 Jul 29;7(35):57011–20. doi: 10.18632/oncotarget.10935 (PMC5302969; doi:10.18632/oncotarget.10935)
Supplement: Supplementary file 1 [file oncotarget-07-57011-s001.pdf]

Functional single nucleotide polymorphisms within the cyclin-dependent kinase inhibitor 2A/2B region affect pancreatic cancer risk

SUPPLEMENTARY TABLE

Supplementary Table S1: List of SNPs and Hardy Weinberg equilibrium values in the populations in the study

SNP	Alleles (M/m)	Call rate	HWE		Minor allele frequency	
			Caucasians	Japanese	Caucasians	Japanese[a]
**rs3731257**	C/T	96.62	0.153	0.638	0.28	0.56
**rs11515**	C/G	98.06	1.000	1.000	0.15	0.01
**rs2518719**	G/A	98.69	0.136	-	0.13	0.00
**rs3731249**	C/T	98.28	0.432	-	0.04	0.00
**rs3731246**	C/G	79.95	0.756	1.000	0.12	0.01
**rs2811708**	T/G	92.75	0.886	0.796	0.26	0.16
**rs3731239**	C/T	97.99	0.222	0.771	0.35	0.14
**rs3731211**	A/T	95.00	0.037	0.215	0.29	0.19
**rs2811710**	T/C	88.48	0.044	0.143	0.37	0.25
**rs3218009**	C/G	99.19	0.031	-	0.10	0.00
**rs3217992**	G/A	98.52	0.607	0.075	0.41	0.57
**rs1063192**	G/A	96.85	0.574	0.142	0.39	0.20
**rs3217986**	C/A	98.52	0.198	0.143	0.08	0.05

[a] Allele frequency of the Caucasian minor allele in the Japanese population.
